# Unraveling the significance of methyltransferase like-3 in the pathogenesis of gastrointestinal tumors: A review

**DOI:** 10.1097/MD.0000000000046857

**Published:** 2026-01-09

**Authors:** Luxi Xiong, Qi Ai, Caijuan Liu, Weiwei Yang, Logen Liu, Guo-Qing Li

**Affiliations:** a Department of Gastroenterology, the Second Affiliated Hospital, Hengyang Medical School, University of South China, Hengyang, China; b Clinical Research Center, the Second Affiliated Hospital, Hengyang Medical School, University of South China, Hengyang, China; c Hunan Provincial Key Laboratory of Basic and Clinical Pharmacological Research on Gastrointestinal Tumors, University of South China, Hengyang, China.

**Keywords:** biomarker, gastrointestinal cancer, Mettl3, N6-methyladenosine (m6A), RNA modification, targeted therapies

## Abstract

In developing countries, gastrointestinal cancer remains a major public health concern due to its high incidence and mortality rates, posing a serious threat to human well-being. N6-methyladenosine, an internal modification found in common eukaryotic RNAs, plays a critical role in the initiation and development of gastrointestinal tumors. Among the key players in N6-methyladenosine formation is methyltransferase-like 3 (Mettl3). This article examines the prognostic implications of Mettl3 expression in gastrointestinal cancers, shedding light on its potential as a biomarker for disease progression and patient outcomes. Understanding the intricate role of Mettl3 in gastrointestinal tumor pathogenesis holds promise for the development of targeted therapies and personalized treatment strategies. Further investigation into the molecular mechanisms and clinical implications of Mettl3 in these cancers will pave the way for more effective interventions and improved patient care in the future.

## 1. Introduction

Gastrointestinal cancer comprises a significant proportion of the cancer burden worldwide, accounting for 26% of the global incidence rate and 35% of all cancer-related deaths. Primary malignancies within the gastrointestinal tract include gastric cancer, liver cancer, esophageal cancer, pancreatic cancer, and colorectal cancer.^[1]^ Genetic changes that regulate epigenetic processes have been found to be drivers of cancer, and unlike genetic events, epigenetic changes are reversible.^[2]^ At present, it has been found that many chemical modifications on human RNA are involved in the development of cancer. RNA posttranscriptional methylation is a field that has been studied more recently, and includes messenger (m)RNA as well as noncoding RNA such as long noncoding (lnc)RNA, circular (circ)RNA, transfer (t)RNA and spliceosome RNA.^[3]^ N6-methyladenosine (m6A) is the most abundant internal modification in eukaryotic mRNA^[4]^ and is a key epitranscriptome modification that controls cell differentiation and pluripotency. Several studies have highlighted the significance of RNA methylation programs in cancer, suggesting their potential as novel and promising approaches for cancer treatment.^[5]^ This process involves 3 major classes of enzymes. The enzyme responsible for catalyzing the methylation of m6A is commonly referred to as the “writer.” Within the m6A writer complex, methyl groups are transferred from the donor substrate, s-adenosylmethionine (SAM), to an adenine base present in the acceptor RNA substrate. This enzymatic activity leads to the addition of m6A modifications to specific RNA molecules, thereby influencing their functional properties in cancer-related processes. Demethylation enzymes are known as “erasers,” and in 2011, the m6A demethylase fat mass and obesity-associated protein was discovered, demonstrating that mRNA methylation is dynamically reversible.^[6,7]^ This discovery was followed by the identification of Alk B homolog 5, also an RNA demethylase.^[8]^ Finally, proteins that recognize m6A modifications are commonly known as “readers.” One such family of readers is the YT512-B homology-domain (YTHD) family. These reader proteins have the ability to selectively bind to dynamic m6A modifications, allowing them to exert influence over the translation status and lifespan of mRNA molecules. By selectively binding to m6A-modified sites, these reader proteins play a crucial role in regulating the functional outcomes of m6A modifications on mRNA molecules.^[9]^

## 2. The functional members of m6A regulation

**Figure 1.**
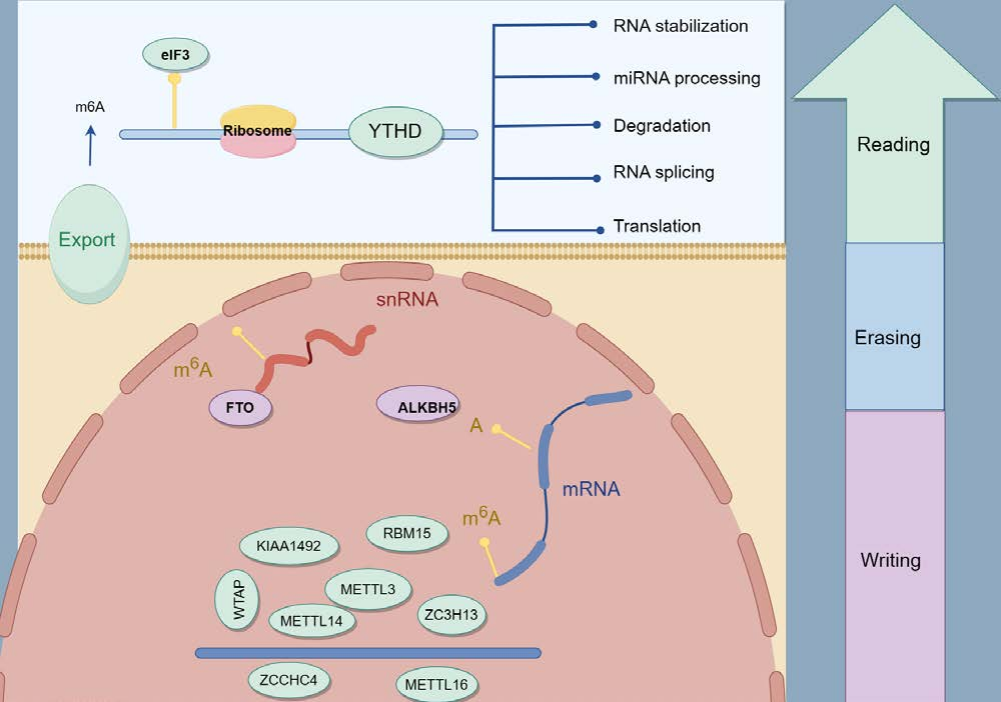
Role of Mettl3 in cancer. Writers refer to the m6A methyltransferases, including methyltransferase-like 3 (METTL3), METTL14, Wilms’ tumor 1-associated protein (WTAP), RNA-binding motif protein 15 (RBM15), vir like m6A methyltransferase associated (VIRMA, also known as KIAA1429), zinc finger CCCH-type containing 13 (ZC3H13), and METTL16. Importantly, m6A can be reversibly removed by m6A eraser proteins, such as fat mass and obesity-associated protein (FTO) and Alk B homolog 5 (ALKBH5). Finally, the YT512-B homology-domain (YTHD) family of reader proteins can recognize m6A sites and perform multiple functions in the nucleus or cytoplasm.

The function of the “writer” in the m6A modification is catalyzed by the m6A methyltransferase complex, which includes methyltransferase-like 3 (Mettl3), methyltransferase-like 14 (Mettl14), and Wilms’ tumor 1-associated protein (WTAP). In addition, vir like m6A methyltransferase associated (also known as KIAA1429), zinc finger CCCH-type containing 13, and RNA-binding motif protein 15 are thought to be part of the m6A methyltransferase complex and are required for m6A methylation.^[10]^ Among these, Mettl14 forms a stable complex by stabilizing the conformation of Mettl3 and recognizing the substrate RNA as structural support to bind with Mettl3. In vitro, both enzymes exhibit limited enzymatic activity when acting independently. However, their activity is significantly enhanced when they are combined in a 1:1 stoichiometric ratio. In the complex formation, Mettl3 serves as the sole methyl donor that binds to SAM and catalyzes the methyl transfer. On the other hand, Mettl14 plays a non-catalytic role by stabilizing the complex and facilitating the recruitment of substrate RNA.^[11–14]^ WTAP itself has no methyltransferase activity, but acts as an adaptor protein that interacts with the Mettl3–Mettl14 complex, thereby significantly affecting the deposition of m6A in cells.^[12]^ Several other proteins assist the function of the Mettl3/Mettl14 complex. Mettl16 was also identified as a human RNA methyltransferase, functioning through the m6A modification of U6 small nuclear (sn)RNA and SAM synthetase pre-mRNA. Moreover, methyltransferase activities have been demonstrated for other proteins, namely Mettl5 and zinc finger CCHC-type containing 4.^[15,16]^ Many other studies have highlighted the pivotal role of Mettl3 in tumorigenesis, tumor progression, and prognosis. This article provides a comprehensive review of the involvement of Mettl3 in gastrointestinal tumors (Fig. 1).

**Figure 2.**
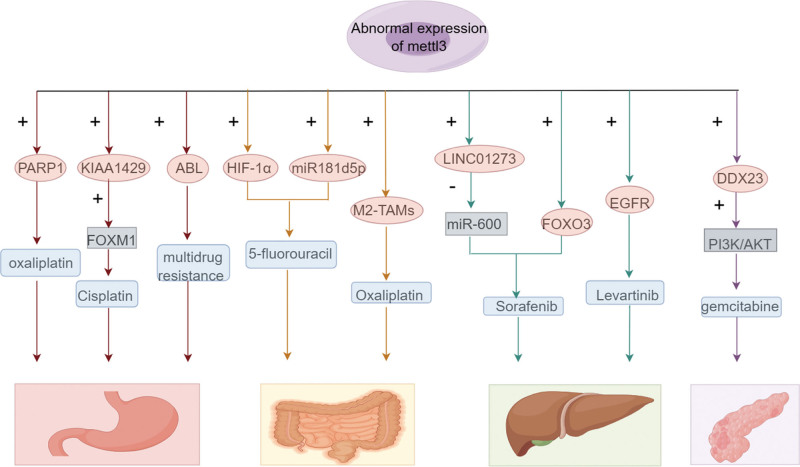
Mettl3 is aberrantly expressed in most tumors. Cancer cells use methyltransferase-like 3 (Mettl3) to promote drug resistance. DDX23 = DEAD-box helicase 23, EGFR = epidermal growth factor receptor, FOXM1 = forkhead box protein M1, FOXO3 = forkhead box O3, PARP1 = poly[ADP-ribose] polymerase 1.

Mettl3 plays a crucial role in regulating the maturation, transportation, and translation of mRNAs. Additionally, it promotes the maturation and activation of noncoding RNAs, including micro (mi)RNAs and long noncoding (lnc)RNAs. Conversely, noncoding RNAs can modulate tumor progression by regulating Mettl3, leading to reduced expression and functionality of Mettl3, which in turn counteracts its tumor-promoting effects.^[17]^ Aberrant expression of Mettl3 is commonly observed in various tumor types. It exerts diverse effects on mRNA translation processes and influences the translation of oncogenes and tumor suppressor genes through multiple mechanisms. Functioning as an oncogene, Mettl3 facilitates the initiation and development of hematopoietic and solid malignancies by depositing m6A modifications on key transcriptional processes.^[16,18]^ Cancer cells exploit Mettl3 to drive drug resistance (Fig. 2). Targeted drugs, such as those targeting MYC, have shown efficacy in cancer treatment, and Mettl3 can serve as an upstream regulator of MYC. Therefore, the development of drugs to inhibit Mettl3 holds great promise as a potential therapeutic approach in cancer treatment.^[19]^

## 3. The role of Mettl3 in digestive system tumors: insights into regulation of mRNA and noncoding RNA maturation, translation, and tumor progression

Many studies have shown that METTL3 is associated with the processes involved in the progression of gastrointestinal cancer, including regulation of mRNA and noncoding RNA, maturation, translation, and tumor progression (Table 1).

**Table 1:** References unveiling the discoveries of methylation-associated factors.

Cancer type	Mechanism	Effect on Mettl3	Biological function	Affected by Mettl3	Effect on drug	References
GC	P300 mediates the transcriptional activation of Mettl3	P300	Enhanced glycolysis in GC, promotion of gastric cancer cell proliferation, angiogenesis, and promotion of gastric cancer metastasis	HDGF		^21^
	HOXA10 enhanced Mettl3 expression	HOXA10	Accelerating EMT progression			^22^
	Silencing HBXIP inhibits Mettl3	HBXIP	Silencing HBXIP inhibiting GC cell proliferation, migration and invasion	MYC		^24^
	Mettl3 enhanced ZMYM1 expression		Accelerating EMT progression	ZMYM1		^23^
	*Mettl3 regulates the stability of PBX1 mRNA through m6A modification*		Enhances the proliferation, migration and invasion ability of AGS cells	PBX1		^26^
	*Mettl3 promote the expression of SPHK2*		Promotes GC cell proliferation, migration and invasion	SPHK2		^27^
	*Mettl3 stabilize DEK mRNA*		Promotes the proliferation and migration of GC cells	DEK		^28^
	*Mettl3 reduces the stability of ADAMTS9 mRNA*		Promote angiogenesis and carcinogenesis in GC	ADAMTS9		^29^
	Ebv-circRPMS1 cooperates with Sam68 to induce Mettl3 transcription	Ebv-circRPMS1	Promoting proliferation, migration and invasion of EBVaGC cells and inhibiting apoptosis			^95^
	BLACAT2 positively regulates Mettl3 by sponging miR-193b-5p	BLACAT2	Promoted GC cells proliferation, migration, invasion, and inhibited apoptosis			^96^
	Positively regulates Mettl3	LINC00240	Promoted proliferation, migration and inhibited apoptosis			^98^
	miR-4429 inhibited Mettl3	miR-4429	Suppressed GC proliferation and enhanced apoptosis	SEC62		^99^
e	miR-1269b inhibits Mettl3	miR-1269b	Suppressed GC proliferation, imigration and invasion			^100^
	Mettl3 promote PARP1 mRNA stability			PARP1	Facilitates oxaliplatin resistance	^30^
	KIAA1429 can link Mettl3, and KIAA1429 stabilizes FOXM1 mRNA	KIAA1429			Facilitated cisplatin resistance	^31^
	Mettl3 maintains ABL stability		Promotes GC cell survival	Abl	May be involved in multidrug resistance	^32^
CRC	Mettl3 enhances SOX2 mRNAs		promotes the occurrence and metastasis of colorectal cancer	SOX2		^34^
	Mettl3 negatively regulates CRB3 expression		Promoted the proliferation, migration, and invasion of CRC cells	CRB3		^35^
	Mettl3 promoted BHLHE41 expression		Inhibit CD8 T cells, thereby promoting CRC	BHLHE41		^36^
	Mettl3 induced GLUT1 translation		Mettl3 promotes CRC initiation and progression	Glut1		^37^
	Mettl3 enhances the stability of PNN RNA		Enhancing CRC cell proliferation, migration and invasiveness	PNN		^38^
	Mettl3 enhancing the expression of MYC		Promote CRC cell proliferation, apoptosis and cell transformation	MYC		^39^
	Mettl3 promotes the maturation of pri-miR-1246		Enhancing metastatic potential	pri-miR-1246		^40^
	Mettl3 promotes the maturation of miR196b		Enhancing metastatic potential	miR-196b		^41^
	Mettl3 promotes the expression of PLAU		Promote angiogenesis and metastasis of CRC	PLAU		^42^
e	Mettl3 maintain LINC00662 and VEGFA high expressions		Promote tumor angiogenesis, proliferation and migration	LINC0066 and VEGFA rna		^43^
	Mettl3 prevent EphA2 mRNA and VEGFA mRNA degradation		Influence vasculature development and support tumor growth	EphA2 and VEGFA		^44^
	Mettl3 suppress the SOCS2		Maintain the tumorigenicity of colon cancer	SOCS2		^45^
	Mettl3 promotes Snail expression		Induces EMT and Malignant Progression in CRC	Snail		^46^
	Mettl3 inhibits p38-ERK pathway		Inhibited CRC cellular growth and metastasis	p-p38 and p-ERK		^47^
	Mettl3 downregulate circ 0000390		Accelerate the proliferation, cell migration, and invasion of CRC cells	Circ_0000390		^101^
	Hsa circ 0000523 regulated Mettl3 expression by suppressing the transcription of miR-let-7b	*hsa_circ_0000523*	Facilitates proliferation, apoptosis and metastasis			^102^
	Mettl3 promote circ_0000677 level		Increase the proliferation, colony formation and drug resistance	Circ_0000677		^103^
	Circ 0001658 positively regulated Mettl3	circ 0001658	Promoted the proliferation of HT29 cells, inhibited apoptosis, and accelerated the cell cycle.			^104^
	Mettl3 suppresses the expression of LINC01559		Enhances CRC cell proliferation and metastasis in vitro	LINC01559		^105^
	LINC01605 bind to Mettl3	LINC01605	Enhances the growth and metastasis of CRC cells	SPTBN2		^106^
	CircLPAR1 competitively inhibits the Mettl3–eIF3h interaction, thereby reducing BRD4 translation		Suppresses colorectal cancer tumorigenesis	circLPAR1		^107^
	Mettl3 increase the transcription of LDHA		Promote glycolysis	LDHA	Promotes 5-FU resistance	^48^
	Mettl3 increased miR-181d-5p expression			pri-mir-181d	Promotes 5-FU resistance	^49^
LC	Mettl3 activates mTORC1 signaling pathway		Enhanced glycolysis	mTOR		^52^
	HBXIP mediates m6A methylation of HIF‐1α mRNA through Mettl3	HBXIP	Promote glycolysis and the malignant biological behaviors, metabolic reprogramming	GLUT1, GLUT3		^53^
	Mettl3 and IGF2BP2 maintained FEN1 expression		Facilitating HCC progression	FEN1		^54^
	SUMOylation of Mettl3 was regulate Snail mRNA homeostasis		Regulate HCC progression	snail		^55^
	MTF1 mRNA transcription is regulated by Mettl3-Mettl14-WTAP complex		Promotes hepatocellular proliferation and angiogenesis	MTF1		^56^
	Mettl3 promoted the expression of ASPM		Promotes LIHC cells growth and metastasis	ASPM		^57^
	Mettl3 positively regulate the malignant phenotype of USP7 in HCC.		Promotes the invasion, migration and proliferation of HCC cells	USP7		^58^
	Mettl3 and YTHDF1 promotes RBM14 expression		Promote the growth and metastasis of tumor	RBM14		^59^
	Mettl3 and IGF2BP2 promote the stability of WWP2		Promotes cell proliferation, colony formation, and glycolysis	WWP2	Inhibits the antitumor effects of doxorubicin	^60^
	Mettl3 represses RDMs expression		Enhances hepatocellular carcinoma progression	RDM1		^61^
	Pinin interacting with Mettl3, which in turn induces snail1 expression	pinin	Promotes invasion and metastasis	snail		^62^
	Mettl3 accelerated IFIT2 mRNA decay		Promotes ICC progression	IFIT2		^63^
	Mettl3 and YTHDF1 enhanced the stability of ANLN		Induce HCC bone metastases	ANLN		^64^
	Mettl3/IGF2BP1 complex enhances the stability of NIFK-AS1 mRNA		Promotes the growth and invasion of HCC cells	NIFK-AS1		^66^
	ILF3-AS1 increased ILF3 m6A level via recruiting Mettl3		Facilitates the proliferation, migration, and invasion of HCC cells	ILF3-AS1		^109^
	Mettl3 lead to downregulation of MEG3		Promotes the proliferation, migration, and invasion of HCC cells	MEG3		^110^
	Mettl3 and YTHDC1 promote Hsa_circ_0058493 intracellular localization from the nucleus to the cytoplasm		Promoted the growth and metastasis of HCC	Hsa_circ_0058493		^111^
	Has_circ_0008583 regulates miR-1301-3p/Mettl3 pathway	Has_circ_000858	Promoted Hep3B cell proliferation, migration, and invasion			^112^
	Mettl3 and ALKBH5 mediate m6 A-modification of circ-CCT3		Downregulation of circ-CCT3 inhibits the growth, metastasis, and angiogenesis of HCC	Circ-CCT3		^113^
	HBx protein upregulated Mettl3 expression	HBx protein	Promoting the progression of HBV-related HCC	circARL3		^114^
	LINC01273 reduce Mettl3 level	LINC01273			Promote sorafenib resistance	^50^
e	Downregulation of Mettl3 mediates the FOXO3 signaling pathway			FOXO3	Downregulation of Mettl3 enhanced sorafenib resistance	^67^
	Mettl3 promoted EGFR translation			EGFR	Promote lenvatinib resistance	^69^
	ERD downregulate Mettl3 expression	ERD	ERD inhibited the migration, invasion, and epithelial–mesenchymal transition of HCC cells			^70^
PC	Mettl3 and IGF2BP3 inhibit the SMS mRNA degradation		Inducing tumor progression and migration in pancreatic cancer	SMS		^73^
	Mettl3 increase the expression of MALAT1		evade immune surveillance and improve the survival ability of PC cells	MALAT1		^74^
	Mettl3 and IGF2BP3 stabilized linc-UROD		Contributes to glycolysis and malignant phenotype of PC cells	linc-UROD		^75^
	Mettl3 enhance LIFR-AS1 mRNA stability		Contribute the development and progression of PC	LIFR-AS1		^76^
	Mettl3 and IGF2BP3 stabilized MYO1C		Promoted the proliferation and migration of PDAC cells	MYO1C		^77^
	Mettl3 and mettl14 participate in the regulation of miR-380-3p		Promotes PC cell proliferation, migration, and EMT in pancreatic cancer	miR-380-3p		^78^
	Mettl3 upregulates PLK1		Create a favorable environment for surrounding fibroblasts for the tumor.	PLK1		^79^
	Mettl3 upregulates the expression of HK2		enhancing glycolysis in PDAC cells and promoting PNI	HK2		^80^
e	Mettl3 modify DDX23 mRNA		promotes cancer cell proliferation and migration	DDX23	Promotes gemcitabine resistance	^82^
	Mettl3 downregulates the levels of DBH-AS1			DBHAS1	Increase PC cell sensitivity to gemcitabine	^109^
ESCC	Mettl3 promote TNFR1 protein translation		Promote tumorigenesis and progression	TNFR1		^86^
	Mettl3 promotes NOTCH1 expression		Promotes esophageal cancer initiation and progression	NOTCH1		^87^
	Mettl3 regulate IFIT2 negatively		Promote ESCC cells proliferation, migration, and invasion	IFIT2		^88^
	Mettl3 promote the expression of COL12A1		Promote ESCC cells proliferation, migration and invasion	COL12A1		^89^
	Mettl3 suppresses APC expression		Enhanced aerobic glycolysis, ESCC cell proliferation, and tumor formation	APC		^90^
	Mettl3 and DGCR8 protein promote the maturity pri-miR-320b		Promotes the proliferation, migration, invasion, and epithelial-mesenchymal transition progression of ESCC cells.	miR-320b		^91^
	Mettl3 enhance GLS2 stability		Promotes cell migration and invasion	GLS2		^92^
	Silencing Caprin-1 decreased the expression of Mettl3 and WTAP	Caprin-1	Silencing Caprin-1 inhibited ESCA cell proliferation and glycolysis			^93^
	Mettl3 increase the stability of EGR1 mRNA		Promotes invasion and metastasis in ESCC	EGR1	Elvitegravir targets Mettl3 to suppress tumor metastasis in ESCC	^94^

5-FU = 5-fluorouracil, ADAMTS9 = ADAM metallopeptidase with thrombospondin type 1 motif 9, ALKBH5 = Alk B homolog 5, ANLN = anillin actin-binding protein, APC = adenomatous polyposis coli, ASPM = abnormal spindle-like microcephaly, BHLHE41 = basic helix-loop-helix family member E41, BRD4 = bromodomain-containing protein 4, COL12A1 = collagen alpha-1(XII) chain, CRB3 = Crumbs3, DDX23 = DEAD-box helicase 23, DGCR8 = DiGeorge syndrome critical region 8, EGFR = epidermal growth factor receptor, EIF3H = factor 3 subunit H, EMT = epithelial-mesenchymal transition, EPHA2 = Ephrin type-A receptor 2, ERD = Resina Draconis, ERK = extracellular signal-regulated kinase, ESCA = esophageal carcinoma, FEN1 = Flap endonuclease-1, FOXM1 = forkhead box protein M1, FOXO3 = forkhead box O3, GLS2 = glutaminase 2, HBXIP = Hepatitis B virus X-interacting protein, HCC = hepatocellular carcinoma, HDGF = hepatoma-derived growth factor, HOXA10 = homeobox A10, IFIT2 = interferon induced protein with tetratricopeptide repeats 2, IGF2BP1 = insulin-like growth factor-2 mRNA-binding protein 1, ILF3-AS1 = ILF3 antisense RNA1, LDHA = lactate dehydrogenase A, LIFR-AS1 = leukemia inhibitory factor receptor antisense RNA 1, LINC00662 = long intergenic nonprotein coding RNA 662, m6A = N6-methyladenosine, MALAT1 = metastasis associated lung adenocarcinoma transcript 1, Mettl14 = methyltransferase-like 14, Mettl3 = methyltransferase-like 3, MTF1 = metal regulatory transcription factor 1, mTOR = mammalian target of rapamycin, NOTCH1 = notch homolog protein 1, PARP1 = poly[ADP-ribose] polymerase 1, PBX1 = pre-B-cell leukemia homeobox 1, PDAC = pancreatic ductal adenocarcinoma, PLAU = urokinase-type plasminogen activator, PLK1 = polo-like kinase 1, PNN = pinin, RDM1 = RAD52 motif-containing protein 1, SAM = s-adenosylmethionine, SMS = Spermine synthase, SOCS2 = suppressor of cytokine signaling 2, SOX2 = sex determining region Y (SRY)-box 2, SPHK2 = sphingosine kinase 2, SPTBN2 = non-erythrocytic 2, SUMO = small ubiquitin-related modifier, TNFR1 = tumor necrosis factor receptor 1, USP7 = ubiquitin-specific peptidase 7, VEGFA = vascular endothelial growth factor A, VIRMA = vir like m6A methyltransferase associated, WTAP = Wilms’ tumor 1-associated protein, WWP2 = WW domain-containing protein 2, YTHD = YT512-B homology-domain, ZMYM1 = zinc finger MYM-type containing 1.

### 3.1. Role of Mettl3 in gastric cancer

Gastric cancer is a highly prevalent disease worldwide, and Mettl3 has emerged as a key player in its pathogenesis. Mettl3 significantly affects the malignant process of gastric cancer through modifications of both mRNA and non-mRNA. Studies have shown that knockout of Mettl3 using CRISPR/Cas9 technology reduces m6A methylation levels in AGS cells (gastric adenocarcinoma cell line), leading to a noticeable inhibition of cell proliferation.^[20]^ Several mRNAs have been identified as promoters of gastric cancer progression due to their interaction with Mettl3. For instance, P300 mediates the transcriptional activation of Mettl3 via epigenetic modification to histone 3, which subsequently maintains the expression of hepatoma-derived growth factor (HDGF) through m6A modifications. This, in turn, activates glucose transporter type 4 and enolase 2 expression to promote glycolysis in gastric cancer cells. The reprogramming of energy metabolism is considered a significant hallmark of cancer. Consequently, upregulation of HDGF expression accelerates the malignant progression of gastric cancer, and includes increased cell proliferation, angiogenesis, and metastasis.^[21]^ Furthermore, Mettl3 promotes epithelial-mesenchymal transition (EMT), a crucial molecular step for distant metastasis of gastric cancer. Studies have revealed that Mettl3 enhances transforming growth factor beta 2 expression by enriching homeobox A10 in the factor beta 2 promoter region, thus activating the TGFβ/Smad pathway and ultimately increasing Mettl3 expression.^[22]^ Additionally, Mettl3-mediated m6A modification enhances zinc finger MYM-type containing 1 expression via an m6A-hur-dependent pathway, leading to the inhibition of epithelial (E)-cadherin transcription through the binding of zinc finger MYM-type containing 1 to the C-terminal-binding protein 1/lysine-specific demethylase 1A/REST corepressor 1 (CtBP/LSD1/CoREST) complex. This consequently promotes EMT and metastasis in gastric cancer.^[23]^ MYC, a well-known oncogene, is a downstream target of Mettl3 in gastric cancer. Silencing hepatitis B x-interacting protein (HBXIP) inhibits Mettl3, which impairs the m6A modification of MYC mRNA, thereby inhibiting gastric cancer cell proliferation, migration, and invasion.^[24,25]^ Moreover, Mettl3 regulates the stability of pre-B-cell leukemia homeobox 1 mRNA through m6A modification, and pre-B-cell leukemia homeobox 1 acts as a transcription factor to induce the expression of GTP cyclohydrolase 1, the rate-limiting enzyme in tetrahydrobiopterin biosynthesis. Increased tetrahydrobiopterin levels enhance the proliferation, migration, and invasion ability of gastric cancer cells.^[26]^ Additionally, Mettl3 promotes the expression of sphingosine kinase 2 in an m6A-YTH N6-methyladenosine RNA binding protein 1 (YTHDF1)-dependent manner. sphingosine kinase 2-mediated phosphorylation of Krüppel-like factor 2 (KLF2) triggers the degradation of KLF2 protein in gastric cancer. Amplification of KLF2 inhibits gastric cancer cell proliferation, migration, and invasion, thereby highlighting the association between KLF2 degradation and poor prognosis in gastric cancer.^[27]^ DEK, considered an oncogenic factor in various cancer processes, is stabilized by Mettl3 through an m6A modification in gastric cancer. DEK, in turn, promotes the proliferation and migration of gastric cancer cells. Conversely, down-regulation of DEK can reverse the effects of Mettl3 on gastric cancer cell proliferation and migration.^[28]^ The downstream target ADAM metallopeptidase with thrombospondin type 1 motif 9 (ADAMTS9) has been discovered to play a crucial role in the context of Mettl3. ADAMTS9 exerts an antitumor effect by inhibiting the phosphoinositide 3-kinase/protein kinase B/mammalian target of rapamycin (PI3K/AKT/mTOR) pathway in gastric cancer. However, the presence of Mettl3 diminishes the stability of ADAMTS9 mRNA through an m6A-YTHDF2-dependent pathway, consequently promoting angiogenesis and carcinogenesis in gastric cancer.^[29]^

When considering drug resistance in gastric cancer, it is important to acknowledge that oxaliplatin is a commonly used first-line treatment for advanced gastric cancer. However, resistance to this drug often leads to treatment failure. Both in vitro and in vivo experiments have demonstrated that CD133^+^ stem-like cells represent a prominent subpopulation within gastric cancer, and the gene poly[ADP-ribose] polymerase 1 (PARP1) plays a central role in mediating resistance to oxaliplatin. Specifically, PARP1 contributes to oxaliplatin resistance through the mechanism of base excision repair. Mettl3, on the other hand, exerts its influence on PARP1 through m6A modification. It targets the 3’-untranslated region of PARP1 mRNA by recruiting YTHDF1, resulting in enhanced stability of PARP1 and consequently facilitating the development of oxaliplatin resistance in gastric cancer cells.^[30]^ Cisplatin is another frequently utilized drug in the treatment of gastric cancer. KIAA1429 is a crucial component of the m6A methyltransferase complex and serves as a link between Mettl3 and the exertion of m6A-related functions. In gastric cancer cells, KIAA1429 plays a role in promoting resistance to cisplatin by ensuring the stability of forkhead box protein M1 mRNA. However, it should be noted that the specific involvement of Mettl3 binding to KIAA1429 in gastric cancer resistance has not been elucidated. Further research is needed to gain a comprehensive understanding of the interplay between Mettl3 and KIAA1429 in the context of gastric cancer resistance.^[31]^ In addition, it was found that apoptotic protease-activating factor 1 (APAF1)-binding lncRNA (ABL) was modified by Mettl3 m6A and recognized by insulin-like growth factor-2 mRNA-binding protein 1 (IGF2BP1), which maintains ABL stability. Then ABL can bind to the WD1/WD2 domain of APAF1, competitively blocking the interaction between cytochrome c and APAF1, ultimately blocking gastric cancer cell apoptosis and potentially contributing to multidrug resistance.^[32]^

### 3.2. The role of Mettl3 in colorectal cancer

Compared to other cancers, colorectal cancer ranks third in terms of incidence, but second in terms of mortality.^[33]^ Mettl3, functioning as an oncogene, exerts its influence by enhancing the stability of the downstream gene sex determining region Y (SRY)-box 2 mRNA through m6A-IGF2BP2 dependent mechanisms. This process ultimately leads to the maintenance of sex determining region Y (SRY)-box 2 expression and contributes to the initiation and metastasis of colorectal cancer.^[34]^ The cell polarity regulator Crumbs3 inhibits proliferation and invasion of colorectal cancer by regulating the Hippo pathway. As a downstream target of Mettl3, Crumbs3 expression is negatively regulated by Mettl3 in an m6A-YTHDF2-dependent manner, thus promoting the proliferation and invasion of colorectal cancer.^[35]^ Mettl3 was also found to promote basic helix-loop-helix family member E41 expression in an m6A-dependent manner, which in turn induces transcription of C-X-C motif chemokine ligand 1. C-X-C motif chemokine ligand 1 is a chemotaxis-stimulating factor that strongly inhibits the antitumor activities of T cells and natural killer cells by recruiting myeloid-derived suppressor cells, thus impairing antitumor immunity and ultimately promoting colorectal cancer.^[36]^ Reprogramming of energy metabolism has emerged as a prominent hallmark of cancer. In the context of colorectal cancer, Mettl3 plays a significant role by inducing the translation of GLUT1 in an m6A-dependent manner. This process promotes glucose uptake and lactate production, leading to the activation of the mammalian target of rapamycin complex 1 (mTORC1) signaling pathway. Inhibition of mTORC1 signaling may potentiate the anticancer effects of Mettl3-mediated silencing in colorectal cancer. Thus, Mettl3 has been identified as a promoter of colorectal cancer through the activation of the m6A-GLUT1-mTORC1 axis. In addition, elevated expression of pinin (PNN) has been observed in colon adenocarcinoma, contributing to enhanced glycolysis in cancer cells and subsequently facilitating their proliferation, migration, and invasiveness. Mettl3, as an upstream regulator of PNN, enhances the stability of PNN RNA through m6A modification, thereby promoting its upregulation.^[37,38]^ It is well known that MYC is an oncogene that plays an important role in cell proliferation, apoptosis and cell transformation. In colon cancer, the MYC oncogene is considered a key target of the wingless (WNT) pathway, and Mettl3 can promote tumor proliferation by enhancing the expression of MYC.^[39]^ During colorectal cancer metastasis, Mettl3 can methylate pri-miR-1246. The anticancer gene sprouty-related EVH1 domain containing 2 is a downstream target of and is negatively regulated by miR-1246. The down-regulation of sprouty-related EVH1 domain containing 2 leads to the inhibition of the mitogen-activated protein kinase (MAPK) pathway, which in turn enhances the metastatic potential of colorectal cancer cells. Additionally, recent research indicates that miR-196b promotes the migration and invasion of colorectal cancer cells. The maturation of miR-196b is dependent on the m6A methylation regulation mediated by Mettl3. Through this regulatory pathway, Mettl3 enhances colorectal cancer metastasis, highlighting the significance of m6A methylation in modulating miRNA-mediated processes in colorectal cancer.^[40,41]^ In terms of promoting tumor angiogenesis, Mettl3 was found to upregulate urokinase-type plasminogen activator mRNA in an m6A-dependent manner, and then participate in the MAPK/extracellular signal-regulated kinase (ERK) pathway to promote angiogenesis and metastasis of colorectal cancer.^[42]^ Mettl3 was also found to promote tumor angiogenesis by simultaneously maintaining the stability of long intergenic nonprotein coding RNA 662 and vascular endothelial growth factor A (VEGFA) RNA.^[43]^ Mettl3 can also target Ephrin type-A receptor 2 and VEGFA to prevent their mRNA degradation through different IGF2BP-dependent mechanisms, and promote the formation of vasculogenic mimicry through PI3K/AKT/mTOR and ERK1/2 signaling pathways, upregulate expression of the vasculogenic mimicry-related gene vimentin to support tumor growth during colorectal cancer tumorigenesis, and is associated with poor prognosis.^[44]^ In addition, Mettl3 negatively regulates suppressor of cytokine signaling 2 (SOCS2) RNA, while SOCS2 expression can inhibit leucine-rich repeat-containing G-protein coupled receptor 5 promoter activity. Low levels of SOCS2 contribute to the maintenance of leucine-rich repeat-containing G-protein coupled receptor 5 expression, a critical factor for the continuous self-renewal of intestinal epithelial cells and a recognized marker of cancer stem cells in colorectal cancer. This down-regulation of SOCS2 promotes the proliferation of colon cancer cells, further emphasizing its role in modulating the growth and maintenance of cancer stem cells in colorectal cancer.^[45]^ Snail is a critical transcriptional factor for EMT, which promotes invasion and proliferation in many cancer types. Importantly, Mettl3 promotes Snail expression through its m6A methylesterase activity, thereby promoting tumor growth and invasion.^[46]^ When Mettl3 acts as a tumor suppressor, it exerts inhibitory effects on the proliferation and migration of colorectal cancer cells, ultimately suppressing tumor growth. These effects are mediated through the regulation of the p38/ERK pathway. Mettl3’s involvement in this pathway leads to the suppression of key signaling molecules, thereby impeding cell proliferation, migration, and the overall progression of colorectal cancer.^[47]^

In the treatment of colon cancer, 5-fluorouracil (5-FU) is an important drug for the treatment of rectal cancer, and resistance to 5-FU usually leads to treatment failure. It has been found that Mettl3 increases the transcription of lactate dehydrogenase A (LDHA) by stabilizing the mRNA of HIF-1α, and triggers the translation of LDHA mRNA by methylation of its coding sequence region and recruitment of YTHDF1. LDHA catalyzes the conversion of pyruvate to lactate, triggering glycolysis and 5-FU resistance.^[48]^ Cancer-associated fibroblast-derived exosomal miR181d5p was also found to inhibit sensitivity to 5-FU by targeting neurocalcin δ, while Mettl3-dependent upregulation of m6A methylation promotes recognition of pri-mir-181d by microprocessor DiGeorge syndrome critical region 8 and processing of mature miR181d5p, promoting miR181d5p formation.^[49]^ Oxaliplatin, a third-generation platinum drug, is widely used to treat colorectal cancer. Tumor-associated macrophages (TAMs) play a critical role in the cross talk between cancer cells and the tumor microenvironment. In solid tumors, TAMs are a distinct subpopulation of macrophages characterized by an M2 phenotype. M2-TAMs have been implicated in promoting resistance to oxaliplatin. This resistance is facilitated by the upregulation of the Mettl3-mediated m6A RNA modification pathway. The increased activity of Mettl3 leads to enhanced modification of RNA molecules. This modification inhibits the action of TNF receptor associated factor 5, a protein involved in necrosis, resulting in the suppression of necrotic cell death. This process ultimately contributes to an increased resistance to oxaliplatin treatment.^[50]^

### 3.3. The role of Mettl3 in liver cancer

Liver cancer is an important cause of cancer-related mortality, and hepatocellular carcinoma (HCC) is the most common form of liver cancer, accounting for 90% of cases.^[51]^ In the context of HCC pathogenesis, it is well-established that tumor cells exhibit elevated glycolysis and increased lactate production under aerobic conditions. This metabolic alteration is considered a hallmark of tumorigenesis and is associated with poor tumor prognosis. Mettl3, in this regard, plays a significant role by activating the mTORC1 signaling pathway and promoting glycolysis in HCC.^[52]^ HBXIP was found to positively regulate Mettl3, and Mettl3-mediated m6A modification of HIF-1α mRNA promoted increased expression of GLUT1 and GLUT3, enhanced glycolysis, and reprogrammed cellular metabolism, thereby promoting the malignancy of HCC.^[53]^ Mettl3-mediated m6A modifies Flap endonuclease-1 (FEN1) mRNA and enhances the stability of FEN1 mRNA by directly recognizing and binding to the m6A site on FEN1 mRNA via IGF2BP2, allowing FEN1 expression to promote HCC growth.^[54]^ Small ubiquitin-related modifier (SUMO) modification, as a posttranslational modification, is considered an important functional regulatory link between cancer growth and metastasis.

In HCC, mitogen stimulation can trigger SUMOylation of Mettl3, and the SUMOylated Mettl3 promotes the progression of HCC by regulating the homeostasis of Snail mRNA and affecting the accumulation of Snail.^[55]^ One study discovered that sulfatide-induced acetylation led to a reduction in the Mettl3-Mettl14-WTAP complex, which in turn resulted in decreased m6A methylation. This reduction in m6A methylation was found to be associated with elevated expression of metal regulatory transcription factor 1 (MTF1). Notably, overexpression of MTF1 was observed to promote cell proliferation and drive tumor progression in HCC. These findings suggest a complex interplay between sulfatide-induced acetylation, the Mettl3–Mettl14–WTAP complex, m6A methylation, and the transcription factor MTF1, highlighting their potential significance in HCC pathogenesis and progression.^[56]^ It was also found that Mettl3 positively regulates abnormal spindle-like microcephaly (ASPM) through m6A modification, whereas overexpression of ASPM promotes the growth and metastasis of hepatoma cells. Conversely, downregulation of ASPM could inhibit the proliferation, migration and invasion of hepatoma cells.^[57]^ Similarly, Mettl3 also regulates the expression of ubiquitin-specific peptidase 7 through m6A methylation, which promotes the invasion, migration and proliferation of HCC cells.^[58]^ In HCC, TAMs play a role in the tumor microenvironment by influencing M1 and M2 polarization. The Mettl3-mediated m6A modification of RBM14 via YTHDF1, which supports the M2 phenotypic polarization of Kupffer cells, promotes the growth and metastasis of the tumor.^[59]^ Mettl3 was also found to mediate WW domain-containing protein 2 (WWP2) m6A modification, which was recognized and bound by IWP2BP2, increasing the stability of WWP2 and leading to WWP2 overexpression. WWP2 activates the AKT signaling pathway, enhances tumor cell growth and glycolytic processes, and inhibits the antitumor effect of Adriamycin.^[60]^ RAD52 motif-containing protein 1 acts as a tumor suppressor in HCC by inhibiting the Ras/Raf/ERK signaling pathway in a P53-dependent manner thereby suppressing tumor growth, while Mettl3 overexpression induces m6A modification of RAD52 motif-containing protein 1 mRNA and suppresses its expression, thereby promoting tumor growth.^[61]^ It was also found that PNN increased the level of m6A modification by interacting with Mettl3, then induced the expression of Snail, which ultimately enhanced the invasive metastasis of HCC.^[62]^ Another finding in the pathogenesis of intrahepatic cholangiocarcinoma is that trimethylation of histone H3 at lysine 4 (H3K4me3) is enriched at the Mettl3 transcriptional start site. H3K4me3 activation-driven Mettl3 transcription promotes intrahepatic cholangiocarcinoma progression through YTHDF2-mediated interferon induced protein with tetratricopeptide repeats 2 (IFIT2) mRNA degradation, and IFIT2 acts as a tumor suppressor.^[63]^ During the process of bone metastasis in HCC, it has been discovered that Mettl3 and YTHDF1 play a role in enhancing the stability of anillin actin-binding protein (ANLN) mRNA through the regulation of m6A modification. Consequently, the nuclear ANLN protein collaborates with specificity protein 1 to form a transcription complex. This complex further augments the kinesin family member 2C/mTORC1 signaling pathway, leading to the promotion of bone metastasis in HCC. These findings highlight the involvement of m6A modification, ANLN, specificity protein 1, and the kinesin family member 2C/mTORC1 pathway in the molecular mechanisms underlying bone metastasis in HCC.^[64]^

Sorafenib is widely used as a first-line drug for treating advanced HCC. However, resistance to sorafenib remains a significant challenge in HCC treatment. Recent studies have identified the role of LINC01273 in promoting resistance to sorafenib by negatively regulating Mettl3 through miR-600 sponge activity. LINC01273 acts as a molecular sponge for miR-600, thereby sequestering miR-600 and preventing its interaction with Mettl3 mRNA. Consequently, the expression of Mettl3 is upregulated, which has been associated with increased resistance to sorafenib. Interestingly, it has been observed that Mettl3 negatively regulates LINC01273 expression, forming a regulatory feedback loop.^[65]^ LncRNA NIFK-AS1 was also found to reduce the injection transporter of sorafenib, making it insensitive to sorafenib treatment in HCC cells.^[66]^ A separate study focused on autophagy, known to be associated with chemo-resistance in human cancers. Forkhead box O3 serves as a key downstream target of Mettl3, and downregulation of Mettl3 mediates the forkhead box O3 signaling pathway to enhance autophagy in HCC, thereby enhancing resistance to sorafenib.^[67]^ Apatinib is a new VEGFR targeting agent, which is 10 times more potent than sorafenib. RG7112 is a small molecule inhibitor that ultimately results in p53 activation. RG7112-sensitized apatinib could demethylate p53 mRNA and reduce p53 mRNA binding to Mettl3, thereby increasing the transcriptional activity of p53 and enhancing its anticancer effect.^[68]^ Lenvatinib is another first-line therapeutic option for advanced HCC. Mettl3 overexpression stimulates the translation efficiency of epidermal growth factor receptor mRNA in HCC cells through m6A modification, which leads to increased epidermal growth factor receptor expression and induces the resistance to lenvatinib.^[69]^ Resina Draconis (ERD), an ethanol extract derived from a herbal source, has demonstrated inhibitory effects on the modification of m6A in Survivin mRNA. This inhibition occurs through the down-regulation of Mettl3 expression and a subsequent decrease in the binding rate of Mettl3 to Survivin mRNA. Survivin, a member of the inhibitor of apoptosis family, plays a crucial role in inhibiting apoptosis and promoting cell survival. By reducing the expression of Survivin protein, the inhibitory effects of ERD can promote apoptosis in HCC cells. Apoptosis, or programmed cell death, is an essential process that helps regulate cell growth and eliminate damaged or abnormal cells.^[70]^

### 3.4. The role of Mettl3 in pancreatic cancer

Pancreatic ductal adenocarcinoma (PDAC) ranks fourth in cancer-related deaths and has a 5-year survival rate of <5%.^[71]^ Therefore, it is necessary to study the pathogenesis and drug resistance mechanisms of pancreatic cancer. Mettl3 plays a crucial role in RNA m6A modification in pancreatic cancer and is involved in the positive regulation of pancreatic cancer cell proliferation and migration. Experimental studies have shown that downregulation of Mettl3 leads to reduced cell proliferation, invasion, and migration in pancreatic cancer. In further investigations using bioinformatics analysis, researchers identified 673 differentially expressed genes associated with the downregulation of Mettl3. These genes were found to be correlated with various cellular components and molecular functions. This suggests that Mettl3 exerts its regulatory effects on pancreatic cancer through the modulation of these genes.^[72]^ Spermine synthase (SMS) has been identified as a downstream target of both Mettl3 and IGF2BP3. These proteins directly impact SMS by inhibiting its mRNA degradation through m6A modification. The overexpression of SMS, in turn, regulates AKT phosphorylation and activates EMT by modulating the conversion of spermine to subspermine. These molecular events ultimately contribute to the progression of pancreatic cancer. The intricate interplay between Mettl3, IGF2BP3, SMS, AKT phosphorylation, and EMT activation sheds light on the underlying mechanisms driving pancreatic cancer progression.^[73]^ It was found that Mettl3 positively regulates the expression of lncRNA metastasis associated lung adenocarcinoma transcript 1 in pancreatic cancer cells, and metastasis associated lung adenocarcinoma transcript 1 increases the expression of programmed death-ligand 1 (PD-L1) in pancreatic cancer cells. PD-L1, as a kind of immunosuppressive factor, helps to form a special tumor microenvironment in pancreatic cancer, which can evade immune surveillance and improve the ability of pancreatic cancer cells to survive.^[74]^ It was also found that linc-UROD was stabilized by Mettl3-induced m6A methylation, then recognized by IGF2BP3. Linc-UROD contributes to glycolysis and the malignant phenotype of PC cells by stabilizing the glycolytic enzymes enolase 1 and pyruvate kinase muscle isozyme.^[75]^ Leukemia inhibitory factor receptor antisense RNA 1 (LIFR-AS1) is a novel cancer-associated lncRNA, transcribed from the LIFR gene in an antisense manner, and Mettl3 was shown to induce LIFR-AS1 m6A to enhance its mRNA stability. LIFR-AS1 can positively regulate VEGFA expression in pancreatic cancer cells via serving as a ceRNA for miR-150-5p, activating VEGFA-mediated AKT/mTOR signaling pathway, and promoting pancreatic cancer progression.^[76]^ CircRNA MYO1C is modified by Mettl3, which induces its circularization, and then interacts with PD-L1 through IGF2BP2 recognition, which promotes the stability of PD-L1 mRNA and promotes the proliferation and migration of tumor cells.^[77]^ In addition, Mettl3 and Mettl14 have been identified as critical regulators of m6A modification on miR-380-3p. Deletion of both Mettl3 and Mettl14 synergistically decreases the expression of miR-380-3p. Notably, the tumor suppressor gene phosphatase and tensin homolog (PTEN) is a downstream target of miR-380-3p. When miR-380-3p is overexpressed, it leads to the degradation of PTEN, resulting in the activation of the AKT pathway. This activation of the AKT pathway contributes to enhanced cell proliferation, migration, and EMT in pancreatic cancer. The interplay between Mettl3, Mettl14, miR-380-3p, PTEN, and the AKT pathway sheds light on the regulatory mechanisms involved in pancreatic cancer progression. Understanding these molecular interactions can provide insights into the development of targeted therapeutic strategies for pancreatic cancer.^[78]^

Mettl3 methylates the 3’-untranslated region of polo-like kinase 1 (PLK1) in pancreatic adenocarcinoma cell lines, and then upregulates PLK1 through IGF2BP2 binding to m6A PLK1. Methylation of PLK1 is essential for the maintenance of the pancreatic cancer cell cycle, and increased Mettl3 may also create a favorable environment for fibroblasts around the tumor and promote tumor growth.^[79]^ Perineural invasion is a typical neuropathological change of pancreatic carcinoma, which is considered a unique metastatic pathway. Research studies have demonstrated that glutamate released from nerve cells can induce calcium influx into PDAC cells via N-methyl-d-aspartate receptors. This calcium influx, in turn, activates the downstream Ca^2+^-dependent protein kinase CaMKII/ERK-MAPK pathway. Activation of this pathway promotes the transcription of the Mettl3 gene, leading to increased Mettl3 expression. Subsequently, Mettl3 upregulates the expression of hexokinase 2 (HK2) through m6A modification in mRNA. This upregulation of HK2 enhances glycolysis in PDAC cells, facilitating the metabolic shift towards increased glucose utilization. Additionally, it promotes perineural invasion, a process by which cancer cells invade along the nerves. The interplay between glutamate signaling, calcium influx, CaMKII/ERK-MAPK pathway activation, Mettl3 expression, HK2 upregulation, enhanced glycolysis, and PNI highlights the complex molecular mechanisms involved in PDAC progression and tumor behavior.^[80]^

In the treatment of pancreatic cancer, previous studies have found that Mettl3 is associated with radiotherapy and chemotherapy resistance of pancreatic cancer. A decrease of Mettl3 can increase the sensitivity to radiotherapy and chemotherapy. Mettl3 may play a role through several key pathways, including the MAPK cascade, ubiquitin-dependent processes, RNA splicing, and regulation of cellular processes.^[81]^ Resistance to gemcitabine, a first-line drug for pancreatic cancer, is a significant contributor to high mortality rates. Mettl3 plays a role in modifying DEAD-box helicase 23 (DDX23) mRNA through m6A modification, leading to increased DDX23 expression. This, in turn, activates the PI3K/AKT signaling pathway, a well-known pathway associated with cancer. Consequently, the activation of this pathway promotes cancer cell proliferation and migration, potentially contributing to gemcitabine resistance. Moreover, studies have demonstrated that silencing Mettl3 sensitizes pancreatic cancer cells to chemotherapy. Mettl3 knockdown partially inhibits the invasive tumor phenotype PDAC by reducing m6A modification of DDX23 mRNA. This suggests that targeting the Mettl3/DDX23 pathway may hold promise as a therapeutic strategy to reverse gemcitabine resistance in PDAC. Understanding the role of Mettl3, DDX23, m6A modification, and the PI3K/Akt pathway in gemcitabine resistance provides insights into potential avenues for overcoming this treatment obstacle in pancreatic cancer. Further research and exploration of targeted therapies aimed at reversing gemcitabine resistance through modulation of the Mettl3/DDX23 pathway are warranted.^[82]^

### 3.5. The role of Mettl3 in esophageal cancer

Esophageal cancer is the sixth leading cause of cancer-related death worldwide,^[83]^ and in China the pathological subtype is predominantly squamous cell carcinoma.^[84]^ Mettl3-mediated m6A methylation plays a key role in esophageal squamous cell carcinogenesis. p21 is known to be a tumor suppressor with important functions in cell cycle and cell proliferation. Mettl3 may regulate the cell cycle through p21 signaling in esophageal. Overexpression of Mettl3 promotes the growth of ESCC cells, while knockdown may lead to G2/M arrest and inhibit tumor growth through p21 signaling.^[85]^ In addition, it was found that Mettl3 could modify tumor necrosis factor receptor 1 (TNFR1) by m6A, and ataxin-2 could promote TNFR1 protein translation in an m6A-dependent manner. The dysregulation of TNFR1 promotes tumorigenesis by activating MAPK and nuclear factor-kappa B pathways downstream of TNFR1.^[86]^ Furthermore, studies have revealed that Mettl3-mediated m6A modification plays a role in regulating the stability of neurogenic locus notch homolog protein 1 (NOTCH1) mRNA, leading to increased expression of the NOTCH1 gene. The Notch signaling pathway is a highly conserved pathway known to regulate various aspects of cancer biology, and its activation has been shown to promote tumorigenesis.^[87]^ By modulating the m6A modification of NOTCH1 mRNA, Mettl3 can influence the stability and expression of NOTCH1, thereby potentially impacting the activation of the Notch signaling pathway. This, in turn, can have implications for cancer initiation and progression. It was also found that Mettl3 negatively regulates the expression of IFIT2 mRNA and protein, and IFIT2 expression inhibits the proliferation and invasion of ESCC cells. Therefore, Mettl3 promotes ESCC cell proliferation, invasion and metastasis by decreasing IFIT2. In addition, Mettl3 and IFIT2 may also mediate the immune response in the development of esophageal cancer.^[88]^ Mettl3 positively regulates collagen alpha-1(XII) chain expression, which in turn regulates the proliferation and metastatic capacity of ESCC cells by mediating RAF/MEK/ERK/MAPK signaling pathway activation. Collagen alpha-1(XII) chain can also restore its oncogenic capacity when METTL3 is knocked out, promoting the development of esophageal cancer.^[89]^ Adenomatous polyposis coli (APC) is a common tumor suppressor that plays a key role in inhibiting the canonical Wnt signaling pathway that controls cell proliferation and differentiation. Mettl3 is responsible for upregulating the m6A modification of APC mRNA. This modification facilitates the recruitment of YTHDF proteins, which promote the degradation of APC mRNA. Consequently, the reduction in APC expression leads to the stabilization of β-catenin and the subsequent modulation of downstream gene expression. This regulatory cascade ultimately promotes aerobic glycolysis and the proliferation of ESCC cells.^[90]^ The extracellular vesicles released by cancer cells have been identified as key mediators of cell-cell communication. In esophageal cancer, miR-320b can be enriched and transferred to human lymphatic endothelial cells through the extracellular vesicles released by ESCC cells. Programmed cell death 4 is a direct target of miR-320b, which inhibits the expression of programmed cell death 4 and stimulates the AKT signaling pathway, thereby promoting the proliferation, migration, invasion, and EMT progression of ESCC cells. During this process, Mettl3 interacts with the DiGeorge syndrome critical region 8 protein and positively modulates the pri-miR-320b maturation process in an m6A-dependent manner, thereby promoting tumor growth.^[91]^

Metastasis is a leading cause of mortality in esophageal cancer, and recent research has identified glutaminase 2 (GLS2) as a critical enzyme involved in glutamine metabolism. Mettl3, through m6A modification, has been found to exert regulatory effects on GLS2 expression. Mettl3 promotes the expression of GLS2 by modifying its mRNA with m6A. The upregulation of GLS2 expression, facilitated by Mettl3-mediated m6A modification, has been observed to enhance the migration and invasion capabilities of ESCC cells. The increased expression of GLS2 may contribute to altered cellular metabolism and other mechanisms involved in cancer progression, ultimately leading to enhanced metastatic potential in ESCC.^[92]^ As an RNA binding protein, cytoplasmic activation/proliferation-associated protein-1 (Caprin-1) participates in a wide range of biological and physiological processes, such as cell proliferation, RNA modification, and the immune response. Silencing Caprin-1 in esophageal cancer has been found to have significant effects. It can inhibit the proliferation of esophageal carcinoma cells by suppressing glycolysis, which is a metabolic process associated with increased glucose utilization. Additionally, downregulating the expression of both Mettl3 and WTAP is involved in slowing the progression of esophageal carcinoma.^[93]^ In addition, in the study of therapeutic mechanism of esophageal cancer, it was found that the zinc-finger transcription factor EGR1 was the target of Mettl3. Mettl3 was able to modify EGR1 with m6A to increase the stability of EGR1 mRNA and promote its expression. It then combines with the Snail promoter sequence to increase Snail expression, thus promoting the progression of esophageal cancer. Elvitegravir, an FDA-approved drug primarily utilized for treating human immunodeficiency virus infection, has been found to have additional implications in cancer research. Specifically, elvitegravir, a component of elvitegravir, binds to Mettl3 and facilitates the degradation of the Mettl3 protein mediated by STIP1 homology and U-Box containing protein 1. This degradation process subsequently leads to the inactivation of the EGR1-Snail signaling pathway, ultimately inhibiting cancer invasion and metastasis. By targeting Mettl3 and promoting its degradation, elvitegravir disrupts the normal functioning of the EGR1-Snail signaling pathway. This signaling pathway is associated with cancer progression and is known to play a role in promoting invasion and metastasis. Therefore, the inhibition of this pathway by elvitegravir contributes to the suppression of cancer cell invasion and metastasis.^[94]^

## 4. Noncoding RNA in m6A regulation

Cellular functions and tumor biology can be influenced by transcriptional regulation, genomic alterations, epigenetic regulation, and posttranslational regulation. Additionally, m6A modifications can be influenced by numerous noncoding RNAs.

First, noncoding RNAs also promote Mettl3 expression. In an Epstein-Barr virus (EBV)-associated gastric cancer, ebv-circRPMS1 (an EBV-encoded cyclic RNA) induced Mettl3 expression by interacting with Sam68 to promote proliferation, migration and invasion of EBVaGC cells and inhibit apoptosis.^[95]^ LncRNAs have been confirmed as new members of the noncoding RNA family, which do not produce proteins but regulate gene expression only at the transcriptional or posttranscriptional level. For example, overexpression of miR-193b-5p inhibited the expression of MettlL3 in gastric cancer cells, while the lncRNA BLACAT2 acted through sponging miR-193b-5p. Mettl3 is a downstream target of miR-193b-5p and is positively regulated by BLACAT2, so BLACAT2 promotes gastric cancer progression by regulating the miR-193b-5p/Mettl3 pathway.^[96]^ In addition, it was found that LINC00240 suppresses the invasion of non-small cell lung cancer cells.^[97]^ Mettl3 was found to be negatively regulated by miR-338-5p, and LINC00240 acts as a sponge for miR-338-5p to control the malignant phenotype of gastric cancer cells by regulating the miR-338-5p/Mettl3 axis. High expression of LINC00240 is beneficial to the malignant phenotype of gastric cancer.^[98]^ miRNA molecules are noncoding RNA with a length of 20 to 22 nucleotides. It has been found that miR-4429, as a miRNA, inhibits Mettl3. Since Mettl3 interacts with the oncogenic factor SEC62 to induce m6A on SEC62 mRNA, thus promoting the stabilizing effect of IGF2BP1 on SEC62 mRNA. Therefore, miR-4429 inhibits the stabilization of SEC62 m6A by targeting Mettl3, thereby inhibiting the progression of gastric cancer. Later, miR-1269b was also found to be a miRNA that could inhibit gastric cancer cell proliferation, migration and invasion by downregulating Mettl3 expression.^[99,100]^

Noncoding RNAs also play a role in the carcinogenesis of colorectal cancer. Overexpression of circular (circ)RNA such as circ_0000390 can inhibit colorectal cancer cell proliferation, migration and invasion by downregulating Notch1. Overexpression of Mettl3 can downregulate circ_0000390, thereby accelerating the malignant activity of colorectal cancer and may serve as a colorectal cancer oncogenic locus.^[101]^ In addition, hsa_circ_0000523 negatively regulates the level of miR-Let 7b in HCT116 cells, which functions as a tumor suppressor in a variety of cancers, and miR-Let 7b binds to Mettl3 to inhibit its expression in colorectal cancer.^[102]^ It was also found that circ_0000677 could positively regulate the expression of ATP binding cassette subfamily C member 1 via its upstream regulator miR-655 and promote tumor development, while Mettl3 could promote circ_0000677 levels by m6A modification, and promote tumor cell proliferation and drug resistance.^[103]^ Recent studies have revealed that circ_0001658 exhibits specific binding to miR-590-5p, leading to the downregulation of its expression. Overexpression of miR-590-5p, in turn, suppresses the growth and migration of colorectal cancer cells. Mettl3, a target of miR-590-5p, demonstrates a negative correlation with miR-590-5p and consequently promotes tumor progression.^[104]^ In addition, it was found that lncRNAs, such as LINC01559, may act as molecular sponges of miR-106b-5p, regulating the expression of PTEN through miR-106b-5p, and the whole axis plays a role in tumor suppression. The overexpression of Mettl3 suppresses the expression of LINC01559, which enhances the proliferation and metastasis of colorectal cancer cells.^[105]^ In addition, the overexpression of LINC01605, facilitated by H3K4me3 and epigenetic modification to histone 3 modifications, can bind to Mettl3 and enhance the m6A modification of non-erythrocytic 2 mRNA. This modification, in turn, promotes the translation of non-erythrocytic 2, consequently stimulating the proliferation, migration, and invasion of colorectal cancer cells.^[106]^ In colorectal cancer tissues, the expression of circLPAR1 is decreased, and this downregulation is also observed in exosomes. Within exosomes, circLPAR1 is capable of interacting with eukaryotic translation initiation factor 3 subunit H (EIF3H). On the other hand, Mettl3 interacts with the EIF3H subunit located at the 5’-end of bromodomain-containing protein 4 mRNA to enhance translation. As a result, circLPAR1 can hinder the interaction between Mettl3 and EIF3H, leading to a reduction in the translation of the oncogene bromodomain-containing protein 4 and subsequently inhibiting tumor growth.^[107]^

LncRNAs are important regulators in the development of HCC, and lncRNA has been found to be a promising prognostic biomarker for gemcitabine-resistant pancreatic cancer. Mettl3 contributes to DBH antisense RNA 1 downregulation. DBH antisense RNA 1 inhibits the ability of pancreatic tumors to grow in vivo and increases the sensitivity of pancreatic cancer cells to gemcitabine by isolating miR-3163 and promoting the upregulation of ubiquitin specific peptidase 44, which is involved in the uptake of gemcitabine.^[108]^ One study found that the Mettl3/IGF2BP1 complex mediated m6A methylation of the lncRNA NIFK-AS1 and enhanced its stability in HCC, whereas high levels of NIFK-AS1 promoted AKT1 expression by sponging miR-637. AKT1 mediates the expression of matrix metalloproteinase 7 and MMP9, which are important metastasis mediators in HCC, thus promoting the growth and invasion of HCC cells.^[66]^ ILF3 antisense RNA1 was found to increase ILF3 m6A levels by recruiting the m6A RNA methyltransferase Mettl3 and enhance the interaction between ILF3 mRNA and the m6A reader IGF2BP1, resulting in stable expression and promoting HCC cell proliferation, migration and invasion.^[109]^ LncRNA MEG3 negatively regulates its downstream gene miR-544b, which positively regulates its target gene B-cell translocation gene (BTG2), and BTG2 inhibits cell proliferation. Mettl3-mediated modification of m6A leads to the downregulation of MEG3, thereby promoting proliferation, migration and invasion of HCC cells.^[110]^

CircRNAs are correlated with the progression and prognosis of HCC. For example, Mettl3 promotes the m6A modification of Hsa_circ_0058493, which promotes its intracellular localization from the nucleus to the cytoplasm in an m6A-dependent manner by binding to YTHDC1, thereby affecting the growth and metastasis of HCC.^[111]^ Has_circ_000858 was also found to bind directly to miR-1301-3p, and Mettl3 is a target of miR-1301-3p. Has_circ_0008583 accelerates the proliferation, migration and invasion of HCC cells by regulating the miR-1301-3p/METTL3 pathway.^[112]^ In addition, circ-CCT3 can be bound by Alk B homolog 5 and Mettl3 and undergo m6A modification, then circ-CCT3 up-regulates the expression of VEGFR1 through sponging miR-378a-3p. As a tyrosine-protein kinase, VEGFR1 plays a key role in cell survival, angiogenesis, cell invasion and migration, and its overexpression promotes the growth and metastasis of HCC.^[113]^ HBV × protein (HBx) is a major driver of HBV-related HCC, which can upregulate the expression of Mettl3 and increase the m6A modification and expression of circARL3. circ-ARL3 was found to sponge miR-1305 and antagonize the inhibition of miR-1305 on a group of target oncogenes, thus promoting the progression of HBV-related HCC.^[114]^

## 5. Conclusions

This comprehensive review highlights the pivotal role of the methyltransferase Mettl3 in mediating m6A modification across gastrointestinal tumors. Mettl3 primarily acts as an oncogene, being tightly regulated by upstream factors and modulating multiple downstream pathways to promote tumorigenesis. In gastric cancer, it enhances malignancy by upregulating oncogenes such as MYC and suppressing tumor suppressors like KLF2. Similarly, in colorectal cancer, Mettl3 drives tumor progression by sustaining HDGF expression and promoting glycolysis. Its oncogenic function in HBV-associated HCC – partly regulated by factors such as HBXIP – further underscores its tumor-promoting potential. In pancreatic cancer, Mettl3 contributes to proliferation, metastasis, and therapeutic resistance, identifying it as a promising biomarker, although its clinical specificity requires further validation. Conversely, its role in esophageal cancer remains largely unexplored, necessitating focused investigation.

Therapeutically, Mettl3 represents a compelling target. Although its involvement in drug resistance poses a major clinical challenge, it also provides opportunities for intervention. For instance, disrupting its interaction with p53 mRNA has been shown to restore chemosensitivity. While no selective Mettl3 inhibitors have yet been developed, natural compounds such as the herbal extract ERD demonstrate potential in suppressing its expression. Future studies should aim to clarify the upstream regulatory networks governing Mettl3, define its context-dependent tumor-suppressive effects, and advance the development of targeted pharmacological inhibitors. Ultimately, a comprehensive understanding of the entire m6A methyltransferase complex – rather than isolated components – will be essential for translating these insights into effective cancer therapies.

## Acknowledgments

We thank Medjaden Inc. for scientific editing of this manuscript.

## Author contributions

**Conceptualization:** Logen Liu.

**Data curation:** Luxi Xiong, Qi Ai, Caijuan Liu, Weiwei Yang.

**Formal analysis:** Luxi Xiong, Qi Ai, Caijuan Liu, Weiwei Yang.

**Funding acquisition:** Guo-Qing Li.

**Writing – original draft:** Luxi Xiong.

**Writing – review & editing:** Logen Liu.
